# Adjunctive albuvirtide is associated with improved immune recovery in treatment-naive patients with advanced HIV disease receiving bictegravir/emtricitabine/tenofovir alafenamide: a retrospective propensity score-matched cohort study

**DOI:** 10.3389/fimmu.2026.1900068

**Published:** 2026-08-10

**Authors:** Yingchun Zhu, Huanxia Liu, Yin Wang, Zongzheng Wang, Lin Cai

**Affiliations:** 1 Clinical Section 2, Department of Infectious Disease, Public Health Clinical Center of Chengdu, Chengdu, China; 2 Clinical Section 1, Department of Infectious Disease, Public Health Clinical Center of Chengdu, Chengdu, China

**Keywords:** advanced HIV disease, albuvirtide, antiretroviral therapy, bictegravir/emtricitabine/tenofovir alafenamide, immune recovery, propensity score matching

## Abstract

**Background:**

Treatment-naive patients with advanced HIV infection may have incomplete immune reconstitution despite effective antiretroviral therapy (ART). We evaluated whether adjunctive albuvirtide (ABT) added to bictegravir/emtricitabine/tenofovir alafenamide (B/F/TAF) was associated with improved 24-week immune recovery and short-term safety.

**Methods:**

This single-center retrospective cohort included adults with baseline CD4+ T-cell counts <200 cells/μL who initiated ABT+B/F/TAF or B/F/TAF alone. Variable-ratio propensity score matching (up to 1:2) yielded 86 patients (33 receiving ABT+B/F/TAF and 53 receiving B/F/TAF). The primary endpoint was a composite of a CD4+ T-cell increase >150 cells/μL and HIV RNA <50 copies/mL at week 24. Secondary outcomes included virological suppression, changes in CD4+ T-cell count and CD4/CD8 ratio, and documented adverse events. A multivariable sensitivity analysis was performed in the complete unmatched cohort (N = 124).

**Results:**

The composite endpoint was achieved more frequently with ABT+B/F/TAF than with B/F/TAF alone (45.5% vs. 22.6%, P = 0.027). The median CD4+ T-cell increase was also greater (165 vs. 106 cells/μL, P = 0.005). HIV RNA <20 copies/mL occurred in 75.8% versus 54.7% of patients, but the difference did not meet the prespecified significance threshold (P = 0.050). In the complete cohort, ABT+B/F/TAF remained associated with immune recovery after multivariable adjustment (adjusted OR = 6.355, 95% CI: 1.539–26.244; P = 0.011). Documented adverse events were similar between groups (54.5% vs. 50.9%, P = 0.732). No Grade ≥3 adverse events, treatment-related adverse events, or adverse-event-related treatment modifications were documented.

**Conclusion:**

Adjunctive ABT was associated with greater short-term immune recovery without an apparent increase in documented adverse events. The retrospective design, limited matched sample, residual confounding, and 24-week follow-up preclude causal inference. These findings require confirmation in prospective randomized trials.

## 1 Introduction

Antiretroviral therapy (ART) has profoundly transformed the clinical trajectory of human immunodeficiency virus (HIV) infection, significantly decreasing both morbidity and mortality globally (1, 2). Current guidelines universally recommend integrase strand transfer inhibitors (INSTIs) (3–5), particularly the single-tablet regimen of bictegravir/emtricitabine/tenofovir alafenamide (B/F/TAF), as the optimal first-line therapy due to its robust antiviral efficacy, favorable safety profile, and high barrier to resistance (6–8). Despite these therapeutic advancements, a considerable proportion of newly diagnosed individuals, reaching up to 61% in China (9), still present with advanced HIV disease characterized by a CD4^+^ T-cell count of less than 200 cells/μL (10). These late-presenting patients face a significantly elevated risk of opportunistic infections and disease-related mortality, primarily driven by severe immune exhaustion and delayed immune reconstitution (11).

Although INSTI-based regimens such as B/F/TAF achieve rapid and durable virological suppression in most treatment-naive patients, a clinically important subgroup with advanced immunodeficiency experiences incomplete or discordant immune recovery despite virological control (12). In these patients, impaired CD4+ T-cell recovery may reflect not only the magnitude and duration of viral replication but also persistent immune activation, chronic inflammation, T-cell exhaustion, impaired thymic output, coinfections, and structural damage to lymphoid tissues. Consequently, interventions that suppress additional stages of the viral life cycle may theoretically provide immunological effects beyond those achieved through intracellular inhibition of reverse transcription and integration alone (13, 14).

Albuvirtide is a long-acting HIV-1 fusion inhibitor that binds to the gp41 region of the viral envelope and prevents membrane fusion before viral entry into susceptible target cells (15, 16). When combined with B/F/TAF, ABT therefore targets a stage of the viral life cycle that is mechanistically distinct from the intracellular actions of bictegravir, emtricitabine, and tenofovir alafenamide. By reducing newly initiated infection events at an early stage, fusion inhibition may decrease continuing antigenic stimulation and help create a more favorable environment for CD4+ T-cell recovery. Nevertheless, this proposed immunological effect remains biologically plausible rather than clinically established, because previous ABT studies have mainly involved treatment-experienced or virologically suppressed populations, and mechanistic biomarkers have rarely been evaluated.

Real-world evidence regarding adjunctive ABT in treatment-naive patients with advanced HIV infection remains limited. We therefore conducted a retrospective propensity score-matched cohort study to compare 24-week immune recovery, virological suppression, and safety outcomes between patients receiving ABT+B/F/TAF and those receiving B/F/TAF alone. We hypothesized that adjunctive ABT would be associated with greater short-term CD4+ T-cell recovery. Given the observational design, the study was intended to generate preliminary evidence rather than establish a causal treatment effect.

## 2 Methods

### 2.1 Study population

This single-center retrospective observational cohort study was conducted at the Chengdu Public Health Clinical Medical Center and was reported in accordance with STROBE principles. Consecutive patients initiating ART between January 2023 and January 2025 were reviewed. Eligible patients were adults aged ≥18 years with confirmed HIV-1 infection, no previous ART exposure, a baseline CD4^+^ T-cell count <200 cells/μL, and initiation of either ABT+B/F/TAF or B/F/TAF alone with available 24-week follow-up data. This retrospective study was approved by the Ethics Committee of Chengdu Public Health Clinical Medical Center (Approval No.2023518), with a waiver of informed consent, and was conducted in accordance with the ethical principles detailed in the Declaration of Helsinki.

### 2.2 Data source and missing data management

The date of ART initiation was defined as baseline. Demographic, clinical, laboratory, treatment, and follow-up data were retrospectively extracted from the electronic medical record system using a standardized data collection form. The recorded baseline variables included age, sex, body mass index (BMI), ethnicity, education level, route of HIV acquisition, marital status, time from diagnosis to treatment initiation, comorbidities, WHO clinical stage, CD4+ T-cell count, CD4/CD8 ratio, and plasma HIV RNA level.

**Figure 1:**
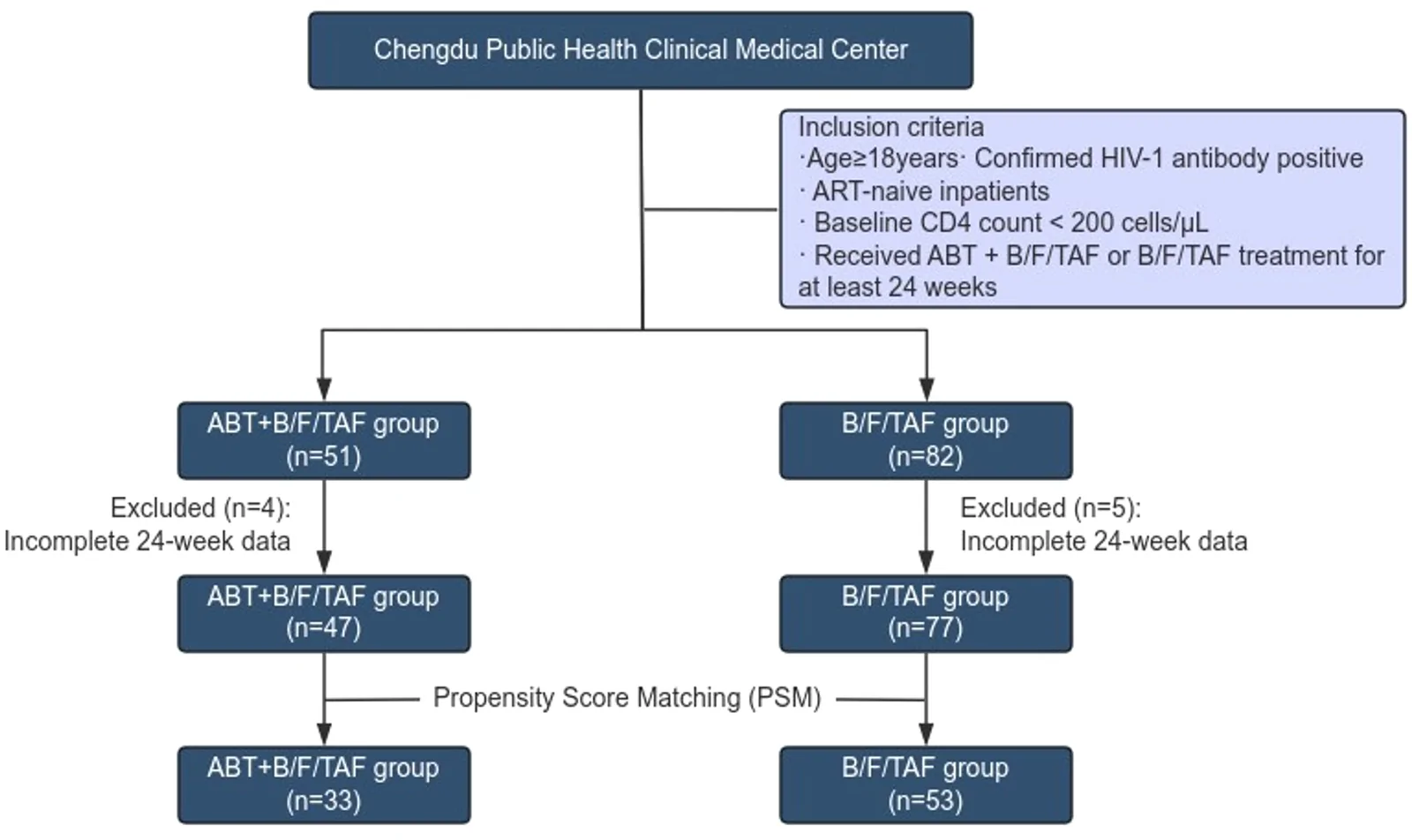
Flowchart of study population selection and treatment allocation.

Data completeness was evaluated before propensity score estimation and outcome analysis. Nine patients without complete week-24 outcome data were excluded during cohort assembly, including four patients in the ABT+B/F/TAF group and five in the B/F/TAF group, as shown in **Figure 1**. Education level was unavailable for 27 patients in the original cohort, all of whom were in the ABT+B/F/TAF group. These observations were retained by coding education as a separate "Unknown" category for descriptive summaries, covariate balance assessment, and the sensitivity analysis; education level was not included in the propensity score model. Missing BMI values were imputed using mean imputation before propensity score estimation and regression analyses. The other variables included in the propensity score model were complete. No multiple imputation was performed.

### 2.3 Outcomes of interest

Immune recovery was defined as a prespecified stringent composite endpoint requiring both virological suppression, defined as HIV RNA <50 copies/mL, and a CD4^+^ T-cell count increase >150 cells/μL at week 24 (17). Secondary outcomes included virological suppression at week 24, defined as HIV RNA <20 copies/mL, changes in CD4+ T-cell count, changes in CD4/CD8 ratio, and safety metrics.

Safety outcomes were retrospectively identified from electronic medical records and available outpatient or inpatient follow-up documentation during the 24-week observation period. Adverse events extracted from the medical records were retrospectively graded according to the Common Terminology Criteria for Adverse Events (CTCAE), version 6.0. The safety analysis included the proportion of patients with at least one reported adverse event, the types and severity of adverse events, investigator-assessed treatment-related adverse events, and treatment modifications due to adverse events, including temporary treatment interruption, delayed administration, dose adjustment, and permanent treatment discontinuation.

### 2.4 Propensity score matching

Propensity score matching (PSM) was used to reduce measured baseline differences between the treatment groups. The probability of receiving ABT+B/F/TAF was estimated for each patient using a multivariable logistic regression model including age, BMI, days to treatment initiation, comorbidities, WHO clinical stage, baseline CD4+ T-cell count, and baseline HIV RNA level. Baseline HIV RNA was entered into the propensity score model on its original continuous scale.

Patients were matched using variable-ratio nearest-neighbor matching, with each patient receiving ABT+B/F/TAF matched to up to two patients receiving B/F/TAF alone. Matching was performed without replacement using variable-ratio nearest-neighbor matching and a caliper width of 0.2 standard deviations of the logit of the propensity score. Patients outside the region of common support or without an eligible comparator within the caliper were not included in the matched cohort.

Covariate balance improved for some variables after matching; however, substantial residualimbalance remained for several covariates. Covariate balance was presented in **Supplementary Table S1** and graphically assessed using a Love plot. Propensity score distributions were examined before and after matching to assess overlap and common support.

### 2.5 Statistical analysis

Continuous variables were summarized as mean ± standard deviation when approximately normally distributed and as median with interquartile range when non-normally distributed. Categorical variables were summarized as frequencies and percentages. Between-group comparisons were performed using the independent-samples t test for approximately normally distributed continuous variables and the Mann–Whitney U test for non-normally distributed continuous variables. Categorical variables were compared using the chi-square test or Fisher’s exact test, as appropriate.

The primary analysis was conducted in the propensity score-matched cohort. To assess whether the principal finding was sensitive to the exclusion of unmatched patients, a prespecified sensitivity analysis was performed in the complete unmatched cohort of 124 patients using logistic regression. The adjusted model included treatment group, age, sex, BMI, education level, route of infection, marital status, days to treatment initiation, comorbidities, baseline CD4+ T-cell count, and log10-transformed baseline HIV RNA. Balance statistics and outcome comparisons in the matched cohort were calculated using the unweighted matched sample.

Results of logistic regression are reported as odds ratios with 95% confidence intervals. All tests were two-sided, and P<0.05 was considered statistically significant. A P value of 0.050 was therefore not considered statistically significant. Analyses of metabolic and laboratory parameters were exploratory, and P values were not adjusted for multiple comparisons.

## 3 Results

### 3.1 Study population and baseline characteristics

The initial analytical cohort included 124 patients, comprising 47 patients receiving ABT+B/F/TAF and 77 receiving B/F/TAF alone. Before PSM, substantial between-group differences were observed in education level, days to treatment initiation, comorbidities, WHO clinical stage, and baseline HIV RNA level (**Table 1**). Propensity score distributions showed a broad region of common support before matching, although the distributions differed between the two treatment groups. Propensity score overlap improved substantially after matching (**Supplementary Figure S2**).

**Table 1:** Baseline demographic and clinical characteristics before and after propensity score matching (matched cohort: ABT+B/F/TAF, n=33; B/F/TAF, n=53).

Characteristics	Before PSM		Statistic	*P value*	After PSM		Statistic	*P value*
	B/F/TAF (N = 77)	ABT + B/F/TAF (N = 47)			B/F/TAF (N = 53)	ABT + B/F/TAF (N = 33)		
Age (years), Mean ± SD	48.99 ± 15.45	48.06 ± 12.21	t=0.369	0.713	49.45 ± 16.56	48.09 ± 12.51	t=0.433	0.666
Sex (%)								
Male	69(89.6)	39(83.0)	χ²=1.142	0.285	47(88.7)	27(81.8)	χ²=0.328	0.567
Female	8(10.4)	8(17.0)			6(11.3)	6(18.2)		
BMI, Mean ± SD	22.36 ± 2.81	22.14 ± 2.37	t=0.470	0.640	21.93 ± 2.50	22.26 ± 2.72	t=-0.585	0.560
Ethnicity(%)								
Han	76(98.7)	44(93.6)	χ²=1.062	0.303	52(98.1)	31(93.9)	χ²=0.178	0.673
Other	1(1.3)	3(6.4)			1(1.9)	2(6.1)		
Education level(%)								
Middle school or below	36(46.8)	9(19.1)	χ²=57.241	<0.001^***^	26(49.1)	7(21.2)	χ²=32.397	<0.001^***^
High school or vocational	9(11.7)	4(8.5)			7(13.2)	4(12.1)		
Bachelor’s degree or above	32(41.6)	7(14.9)			20(37.7)	6(18.2)		
Unknown	0(0)	27(57.4)			0(0.0)	16(48.5)		
Route of infection(%)								
Uncertain	9(11.7)	3(6.4)	χ²=3.369	0.338	6(11.3)	2(6.1)	χ²=3.637	0.303
Injecting drug use	0(0.0)	1(2.1)			0(0.0)	1(3.0)		
Homosexual	23(29.9)	11(23.4)			17(32.1)	7(21.2)		
Heterosexual	45(58.4)	32(68.1)			30(56.6)	23(69.7)
Marital status(%)								
Married	45(58.4)	27(57.4)	χ²=0.012	0.913	29(54.7)	19(57.6)	χ²=0.067	0.795
Unmarried/Divorced/Widowed	32(41.6)	20(42.6)			24(45.3)	14(42.4)		
Days to treatment initiation(%)								
>7.0	55(71.4)	24(51.1)	χ²=5.235	0.022^*^	34(64.2)	21(63.6)	χ²=0.002	0.961
≤7.0	22(28.6)	23(48.9)			19(35.8)	12(36.4)		
Comorbidities(%)								
0	37(48.1)	9(19.1)	χ²=10.448	0.001^***^	17(32.1)	9(27.3)	χ²=0.222	0.637
1	40(51.9)	38(80.9)			36(67.9)	24(72.7)		
WHO clinical stage(%)								
2	27(35.1)	10(21.3)	χ²=6.284	0.043^*^	15(28.3)	7(21.2)	χ²=0.642	0.725
3	33(42.9)	17(36.2)			22(41.5)	14(42.4)		
4	17 (22.1)	20 (42.6)			16(30.2)	12(36.4)		
Baseline CD4, Median (Q1,Q3)	105.00 (63.00, 146.00)	94.00 (28.00, 137.00)	z=1.543	0.123	105.00(65.00,142.00)	94.00(30.00,140.00)	z=0.950	0.342
Baseline CD4/CD8, Median (Q1,Q3)	0.15 (0.09, 0.23)	0.14 (0.06, 0.27)	z=0.682	0.495	0.13(0.09,0.22)	0.14(0.06,0.26)	z=0.099	0.921
Baseline HIV RNA, Median (Q1,Q3), copies/mL	126000.00 (40600.00, 449000.00)	275000.00 (88850.00, 1080000.00)	z=-2.549	0.011	126000.00(55700.00,476000.00)	232000.00(88600.00,1040000.00)	z=-0.305	0.760
Baseline HIV RNA group (%)								
<100,000	31 (40.3)	12 (25.5)	χ²=2.790	0.090	19(35.8)	10(30.3)	χ²=0.280	0.597
≥100,000	46 (59.7)	35 (74.5)			34 (64.2)	23 (69.7)		

B/F/TAF denotes Bictegravir/Emtricitabine/Tenofovir Alafenamide. Percentages for ‘Education level’ account for the ‘Unknown’ category (N = 27) recorded in the original dataset, ensuring the cumulative total reaches 100%. *P < 0.05, **P < 0.01, and ***P < 0.001.

Variable-ratio PSM resulted in a matched cohort of 86 patients, including 33 patients in the ABT+B/F/TAF group and 53 patients in the B/F/TAF group. After matching, covariate balance improved for some baseline characteristics; however, 9 of the 13 reported baseline characteristics had at least one level or measure with an absolute SMD ≥0.10. The largest residual imbalances involved education level, baseline HIV RNA, route of infection, baseline CD4+ T-cell count, ethnicity, and sex. In particular, the "Unknown" education category remained confined to the ABT+B/F/TAF group. These residual imbalances were considered when interpreting the matched analysis.

### 3.2 Immunological responses and clinical outcomes

At week 24, the study-defined composite immune recovery endpoint was achieved by 15 of 33 patients in the ABT+B/F/TAF group and 12 of 53 patients in the B/F/TAF group, corresponding to rates of 45.5% and 22.6%, respectively (P = 0.027) (**Table 2**). This represented an absolute between-group difference of 22.9 percentage points. The median increase in CD4+ T-cell count was 165 cells/μL (IQR: 108–194) in the ABT+B/F/TAF group and 106 cells/μL (IQR: 71–148) in the B/F/TAF group (P = 0.005), corresponding to a 59 cells/μL greater median increase with adjunctive ABT.

**Table 2:** Virological and immunological outcomes at week 24 in the propensity score-matched cohort (ABT+B/F/TAF, n=33; B/F/TAF, n=53).

Variables	Total (N = 86)	B/F/TAF (N = 53)	ABT + B/F/TAF (N = 33)	Statistic	*P value*
HIV RNA (copies/mL), Median (Q1,Q3)					
Baseline	164000.00 (70600.00, 619250.00)	126000.00 (55700.00, 476000.00)	232000.00 (88600.00, 1040000.00)	*Z* = -0.305	0.760
week 24	20.00 (20.00, 37.60)	20.00 (20.00, 42.10)	20.00 (20.00, 20.00)	*Z* = 1.734	0.083
ΔHIV RNA (copies/mL), Median (Q1,Q3)					
Week 24 change from baseline	163980.00 (69705.00, 629975.00)	125980.00 (53997.35, 477980.00)	231980.00 (80330.00, 1079955.95)	*Z* = -1.496	0.135
Virological suppression rate					
HIV RNA < 20 copies/mL, n(%)	54/86(62.8)	29/53(54.7)	25/33(75.8)	χ²=3.854	0.050
Immune recovery, n(%)	27/86(31.4)	12/53(22.6)	15/33(45.5)	χ²=4.914	0.027^*^
CD4, Median (Q1,Q3)					
Baseline	100.00 (60.25, 141.75)	105.00 (65.00, 142.00)	94.00 (30.00, 140.00)	*Z=*0.950	0.342
week 24	224.50 (153.25, 297.00)	210.00 (152.00, 280.00)	260.00 (157.00, 330.00)	*Z=*-1.199	0.231
Change from baseline	119.50 (82.75, 175.75)	106.00 (71.00, 148.00)	165.00 (108.00, 194.00)	*Z=*-2.793	0.005^**^
CD4/CD8, Median (Q1,Q3)					
Baseline	0.14 (0.08, 0.23)	0.13 (0.09, 0.22)	0.14 (0.06, 0.26)	*Z=*0.099	0.921
week 24	0.26 (0.17, 0.43)	0.25 (0.16, 0.42)	0.30 (0.19, 0.44)	*Z=*-0.569	0.570
Change from baseline	0.10 (0.06, 0.22)	0.09 (0.05, 0.18)	0.12 (0.06, 0.22)	*Z=*-0.573	0.567

HIV RNA levels are reported in copies/mL. A value of 20 copies/mL represents the lower limit of detection of the assay. In this table, virological suppression was defined as HIV RNA <20 copies/mL, whereas the study-defined composite immune recovery endpoint used HIV RNA <50 copies/mL together with a CD4+ T-cell increase >150 cells/μL. *P < 0.05 and **P < 0.01.

At week 24, median CD4+ T-cell counts had increased in both groups. The median week-24 CD4+ T-cell count was 260 cells/μL (IQR: 157–330) in the ABT+B/F/TAF group and 210 cells/μL (IQR: 152–280) in the B/F/TAF group, although the between-group difference in absolute week-24 counts was not statistically significant (P = 0.231). The CD4/CD8 ratio also increased in both groups, with no significant difference in the change from baseline between the ABT+B/F/TAF and B/F/TAF groups (median change: 0.12 vs. 0.09; P = 0.567). No deaths or newly diagnosed opportunistic infections were documented during the 24-week follow-up.

### 3.3 Virological suppression

At week 24, HIV RNA <20 copies/mL was achieved by 25 of 33 patients receiving ABT+B/F/TAF and 29 of 53 patients receiving B/F/TAF alone. The corresponding suppression rates were 75.8% and 54.7%, representing an absolute difference of 21.1 percentage points. However, the between-group comparison yielded P = 0.050 and therefore did not meet the prespecified threshold of P<0.05. This finding was interpreted as a numerical difference rather than statistically confirmed superiority.

The median decline in HIV RNA from baseline was 231,980 copies/mL (IQR: 80,330–1,079,955.95) in the ABT+B/F/TAF group and 125,980 copies/mL (IQR: 53,997.35–477,980) in the B/F/TAF group, with no statistically significant difference between groups (P = 0.135).

### 3.4 Association of ART regimens with immune recovery

A prespecified sensitivity analysis was performed in the complete unmatched cohort of 124 patients to evaluate whether the association observed in the matched cohort was sensitive to the exclusion of unmatched patients (**Table 3**). In the unadjusted model, ABT+B/F/TAF was associated with higher odds of achieving the composite immune recovery endpoint than B/F/TAF alone (OR = 2.521, 95% CI: 1.148–5.535; P = 0.021).

**Table 3:** Unadjusted and multivariable-adjusted logistic regression analyses of factors associated with immune recovery in the complete unmatched cohort (N = 124).

Variables	N	β	SE	z value	OR (95%CI)	*P value*
Unadjusted						
Group	124					
B/F/TAF	77				Ref.	
ABT + B/F/TAF	47	0.925	0.401	2.305	2.521 (1.148, 5.535)	0.021^*^
Adjusted						
Group	124					
B/F/TAF	77				Ref.	
ABT+B/F/TAF	47	1.849	0.724	2.556	6.355(1.539, 26.244)	0.011^*^
Age	124	-0.025	0.031	-0.816	0.975(0.918, 1.036)	0.414
Sex	124					
Male	108				Ref.	
Female	16	0.051	1.220	0.042	1.052(0.096, 11.507)	0.967
BMI	124	0.144	0.117	1.227	1.155(0.918, 1.454)	0.220
Education level	124					
Middle school or below	45				Ref.	
High school or vocational	13	0.734	1.028	0.714	2.084(0.278, 15.642)	0.475
Bachelor’s degree or above	39	1.496	0.881	1.698	4.464(0.794, 25.100)	0.089
Route of infection	124					
Uncertain	12				Ref.	
Homosexual	34	-0.070	1.004	-0.070	0.932(0.130, 6.667)	0.944
Heterosexual	77	-0.113	0.978	-0.116	0.893(0.131, 6.068)	0.908
Marital status	124					
Married	72				Ref.	
Unmarried/Divorced/Widowed	52	0.163	0.811	0.200	1.176(0.240, 5.767)	0.841
Days to treatment initiation	124					
>7.0	79				Ref.	
≤7.0	45	0.787	0.614	1.283	2.198 (0.660, 7.317)	0.199
Comorbidities	124					
0	46				Ref.	
1	78	-1.203	1.188	-1.012	0.300 (0.029, 3.085)	0.312
Baseline CD4	124	0.017	0.008	2.027	1.017 (1.001, 1.034)	0.043^*^
Baseline HIV RNA, log10 copies/mL	124	0.215	0.364	0.591	1.240 (0.607, 2.533)	0.555

*P < 0.05.

After adjustment for age, sex, BMI, education level, route of infection, marital status, days to treatment initiation, comorbidities, baseline CD4+ T-cell count, and log10-transformed baseline HIV RNA, the association remained statistically significant (adjusted OR = 6.355, 95% CI: 1.539–26.244; P = 0.011). Higher baseline CD4+ T-cell count was also associated with increased odds of immune recovery (adjusted OR per 1-cell/μL increase=1.017, 95% CI: 1.001–1.034; P = 0.043). The wide confidence interval for the treatment effect indicates limited estimate precision and should be considered when interpreting the magnitude of the association.

### 3.5 Safety and adverse events

During the 24-week observation period, 18 of 33 patients (54.5%) in the ABT+B/F/TAF group and 27 of 53 patients (50.9%) in the B/F/TAF group had at least one adverse event documented in the available medical records (P = 0.732). Most reported adverse events were mild to moderate according to CTCAE version 6.0. No Grade ≥3 AEs were documented in either group. No adverse events were judged to be treatment-related. In addition, no temporary treatment interruptions, delayed administrations, dose adjustments, or permanent treatment discontinuations due to adverse events were documented in either group (**Table 4**).

**Table 4:** Reported adverse events during the 24-week follow-up in the propensity score-matched cohort (ABT+B/F/TAF, n=33; B/F/TAF, n=53).

Variables	ABT + B/F/TAF (N = 33), n (%)	B/F/TAF (N = 53), n (%)	*P value*
Overall Summary			
Patients with ≥1 reported AE, n (%)	18 (54.55%)	27 (50.94%)	0.732
Grade ≥3 AEs, n (%)	0 (0.00)	0 (0.00)	–
Treatment-related AEs, n (%)	0 (0.00)	0 (0.00)	–
Temporary treatment interruption due to AE, n (%)	0 (0.00)	0 (0.00)	–
Delayed administration due to AE, n (%)	0 (0.00)	0 (0.00)	–
Dose adjustment due to AE, n (%)	0 (0.00)	0 (0.00)	–
Permanent treatment discontinuation due to AE, n (%)	0 (0.00)	0 (0.00)	–
Gastrointestinal system			
Nausea	4 (12.12%) [3 Grade 1, 1 Grade 2]	5 (9.43%) [4 Grade 1, 1 Grade 2]	0.694
Diarrhea	3 (9.09%) [All Grade 1]	4 (7.55%) [All Grade 1]	0.786
Vomiting	2 (6.06%) [1 Grade 1, 1 Grade 2]	3 (5.66%) [2 Grade 1, 1 Grade 2]	0.940
Abdominal pain	1 (3.03%) [All Grade 1]	2 (3.77%) [All Grade 1]	0.861
**Nervous system**			
Headache	5 (15.15%) [4 Grade 1, 1 Grade 2]	6 (11.32%) [5 Grade 1, 1 Grade 2]	0.540
Dizziness	3 (9.09%) [All Grade 1]	4 (7.55%) [All Grade 1]	0.790
Insomnia	2 (6.06%) [All Grade 1]	3 (5.66%) [All Grade 1]	0.940
Blood and lymphatic system			
Leukopenia	2 (6.06%) [1 Grade 1, 1 Grade 2]	3 (5.66%) [2 Grade 1, 1 Grade 2]	0.941
Thrombocytopenia	1 (3.03%) [All Grade 1]	2 (3.77%) [All Grade 1]	0.857
Hepatic and renal function			
Elevated transaminases	3 (9.09%) [2 Grade 1, 1 Grade 2]	4 (7.55%) [3 Grade 1, 1 Grade 2]	0.792
Mildly elevated creatinine	1 (3.03%) [All Grade 1]	1 (1.89%) [All Grade 1]	0.740
Other AEs.			
Rash	2 (6.06%) [All Grade 1]	3 (5.66%) [All Grade 1]	0.940
Fatigue	4 (12.12%) [All Grade 1]	5 (9.43%) [All Grade 1]	0.693

Values represent the number and percentage of patients with at least one documented event. Individual patients may have experienced more than one type of adverse event. Adverse events were retrospectively extracted from routine medical records and graded according to CTCAE version 6.0. No Grade ≥3 AEs, treatment-related adverse events, temporary treatment interruptions, delayed administrations, dose adjustments, or permanent treatment discontinuations due to adverse events were documented in either group.

### 3.6 Metabolic and laboratory parameter changes

At baseline, the two groups exhibited comparable levels in most metabolic parameters, with the exception of triglycerides (TG) and total bilirubin (TBIL) (**Table 5**). In exploratory analyses of week-24 changes, the ABT+B/F/TAF group showed smaller increases in TC and LDL-C than the B/F/TAF group (TC: *P* = 0.009; LDL-C: *P* = 0.010). These findings were not adjusted for multiple comparisons and should therefore be interpreted descriptively. Changes in fasting glucose, hemoglobin, creatinine, ALT, AST, and TBIL were generally comparable between cohorts. The between-group comparison for TBIL change yielded P = 0.050 and therefore did not meet the prespecified threshold for statistical significance.

**Table 5:** Baseline values and week-24 changes in metabolic and laboratory parameters in the propensity score-matched cohort (ABT+B/F/TAF, n=33; B/F/TAF, n=53).

Parameters	Baseline		*P value*	Change at week 24		*P value*
	ABT + B/F/TAF (N = 33)	B/F/TAF (N = 53)		ABT + B/F/TAF (N = 33)	B/F/TAF (N = 53)	
TG (mmol/L)	1.83 (1.27, 2.40)	1.51 (1.04, 1.75)	0.020^*^	0.07 (-0.58, 0.60)	0.22 (-0.35, 0.98)	0.260
TC (mmol/L)	3.94 (3.17, 4.19)	3.94 (3.50, 4.28)	0.980	0.19 (-0.07, 0.84)	0.89 (0.20, 1.47)	0.009^**^
HDL-C (mmol/L)	0.98 (0.87, 1.14)	0.91 (0.77, 1.06)	0.140	0.27 (0.02, 0.37)	0.22 (0.07, 0.48)	0.690
LDL-C (mmol/L)	2.26 (1.67, 2.76)	2.50 (2.07, 2.87)	0.100	-0.05 (-0.32, 0.56)	0.43 (-0.05, 0.98)	0.010^*^
Glu (mmol/L)	5.00 (4.61, 5.77)	5.43 (4.92, 6.16)	0.050	0.39 (-0.41, 1.07)	0.20 (-0.35, 0.70)	0.510
Hb (g/L)	127.00 (107.00, 145.00)	135.00 (121.50, 146.00)	0.110	14.00 (4.00, 28.22)	11.00 (0.00, 23.00)	0.290
Cr (μmol/L)	74.00 (60.50, 85.00)	68.53 (59.00, 77.50)	0.140	9.00 (1.50, 13.00)	13.00 (1.50, 20.00)	0.189
ALT (U/L)	34.00 (20.00, 44.50)	30.00 (21.00, 48.50)	0.970	-5.00 (-26.40, 9.02)	-4.00 (-14.00, 8.00)	0.542
AST (U/L)	34.00 (24.00, 53.00)	29.00 (23.50, 34.00)	0.090	-7.00 (-21.50, 2.08)	-6.00 (-10.00, 2.50)	0.240
TBIL (μmol/L)	8.22 (5.60, 10.50)	11.67 (7.55, 12.75)	0.020^*^	3.41 (-0.95, 6.65)	-0.30 (-2.60, 3.45)	0.050

Values are presented as median (Q1, Q3). The analyses of metabolic and laboratory parameters were exploratory. P values were not adjusted for multiple comparisons and should be interpreted descriptively. *P < 0.05 and **P < 0.01.

## 4 Discussion

In this retrospective propensity score-matched cohort of treatment-naive patients with advanced HIV infection, adjunctive ABT was associated with a higher probability of achieving the prespecified composite immune recovery endpoint at week 24. The absolute difference in immune recovery was 22.9 percentage points, and the median increase in CD4+ T-cell count was 59 cells/μL greater in the ABT+B/F/TAF group than in the B/F/TAF group. These differences may be clinically meaningful in patients starting ART with CD4+ T-cell counts below 200 cells/μL, because even moderate early gains in CD4+ T-cell count may reduce the duration of severe immunodeficiency. However, no deaths or new opportunistic infections occurred during the short follow-up, and the study was not powered to demonstrate whether the observed immunological difference translated into fewer clinical events.

Virological suppression at HIV RNA <20 copies/mL was numerically more frequent in the ABT+B/F/TAF group, but the difference did not meet the prespecified threshold for statistical significance. This result should not be interpreted as evidence of virological superiority. A recent retrospective cohort comparing TLD and TLE in ART-naive adults similarly highlighted that first-line regimen selection may influence virological, immunological, and tolerability outcomes (18). However, direct comparisons across studies are limited by differences in baseline immune status, treatment regimens, viral-load thresholds, and follow-up duration. Likewise, comparisons with phase 3 B/F/TAF trials should be interpreted cautiously because those trials generally included less immunocompromised populations and assessed HIV RNA <50 copies/mL at later time points.

Safety findings were broadly comparable between the two treatment groups during the 24-week follow-up. Although approximately half of the patients in each group had at least one reported adverse event, these events were predominantly mild to moderate according to CTCAE version 6.0. No Grade ≥3 AEs were identified, and no adverse events were judged to be treatment-related. In addition, no temporary treatment interruptions, delayed administrations, dose adjustments, or permanent treatment discontinuations due to adverse events were documented. These findings suggest that adjunctive ABT was not associated with an apparent short-term safety penalty in this cohort.

Exploratory laboratory analyses showed smaller increases in total cholesterol and LDL-C in the ABT+B/F/TAF group. These observations should not be interpreted as evidence that ABT improves lipid metabolism. The analyses were not adjusted for multiple comparisons, baseline triglyceride and bilirubin levels differed between groups, and potentially relevant factors such as lipid-lowering therapy, dietary intake, body-weight change, and systemic inflammation were not comprehensively captured. The lipid findings should therefore be considered hypothesis-generating and require prospective confirmation.

The observed association with immune recovery cannot be explained solely by the reduction in plasma HIV RNA. Incomplete immune reconstitution despite effective ART is multifactorial and has been associated with persistent immune activation, chronic inflammatory signaling, impaired thymic output, T-cell exhaustion, coinfections, and damage to lymphoid tissue architecture (12). ABT inhibits gp41-mediated membrane fusion before HIV enters susceptible target cells, whereas the components of B/F/TAF inhibit intracellular integration and reverse transcription. These complementary actions may reduce newly initiated infection events and continuing antigenic stimulation during the early phase of treatment, thereby creating a more favorable environment for CD4+ T-cell expansion and survival.

Nevertheless, this mechanism remains speculative. The present study did not assess immune activation or inflammatory biomarkers such as interleukin-6, soluble CD14, D-dimer, activated or exhausted T-cell phenotypes, thymic output, or reservoir-related measurements. Moreover, previous ART-intensification strategies have not consistently improved immune reconstitution in patients with established immune non-response. The findings therefore support additional mechanistic investigation but do not establish that ABT directly reduces immune activation or restores T-cell function.

The potential clinical importance of improved immune recovery also extends beyond short-term virological outcomes. As survival among people with HIV continues to improve, non-AIDS comorbidities have become increasingly important determinants of long-term health. A recent multicenter study reported a substantial burden of echocardiographic abnormalities among adults with HIV and identified advanced immunosuppression and ART-naive status as factors associated with dilated cardiomyopathy (19). Although the present study did not evaluate cardiovascular outcomes, these observations emphasize that early immune restoration and comprehensive long-term risk management should be considered complementary objectives of HIV care.

Several limitations should be considered. First, this was a single-center retrospective observational study in which treatment allocation was determined clinically rather than randomly. PSM reduced some measured baseline differences but did not eliminate substantial residual imbalance, particularly for education level.

Second, the matched sample was relatively small, with 33 patients in the ABT+B/F/TAF group and 53 in the B/F/TAF group. Some eligible patients were excluded because they fell outside the region of common support or lacked an eligible match within the caliper, which reduced statistical power and may limit generalizability to the complete treatment population. Although the sensitivity analysis in the unmatched cohort yielded a directionally consistent association, the wide confidence interval around the adjusted treatment estimate indicates substantial uncertainty regarding the magnitude of the effect.

Third, matching left substantial residual baseline imbalance, particularly for education level, baseline HIV RNA, route of infection, baseline CD4+ T-cell count, ethnicity, and sex; residual confounding therefore cannot be excluded. Coding missing education records as an “Unknown” category preserved sample size but may not fully address differential missingness. Fourth, adverse events were retrospectively identified from routine medical records rather than prospectively collected using a trial-specific safety protocol. Although adverse events were graded according to CTCAE version 6.0 and no treatment-related adverse events or treatment modifications were identified, under-ascertainment of mild adverse events remains possible in a retrospective study.

Fifth, follow-up was limited to 24 weeks. The study therefore could not determine whether the early CD4+ T-cell difference was sustained or translated into durable virological control, fewer opportunistic infections, reduced non-AIDS morbidity, or improved survival. Finally, immune activation, inflammatory, exhaustion, and viral-reservoir biomarkers were unavailable, preventing direct evaluation of the proposed biological mechanism.

Despite these limitations, the consistent direction of the matched and multivariable-adjusted analyses provides a rationale for prospective evaluation. Future multicenter randomized controlled trials should compare B/F/TAF with and without adjunctive ABT using prespecified virological and immunological endpoints, standardized adverse-event assessment, adherence monitoring, and immune activation biomarkers. Longer follow-up will be required to determine whether early CD4+ T-cell gains are sustained and associated with clinically meaningful reductions in AIDS-related and non-AIDS events.

In conclusion, adjunctive ABT was associated with greater short-term immune recovery among treatment-naive patients with advanced HIV infection receiving B/F/TAF. The findings should be considered preliminary and hypothesis-generating because of the retrospective design, limited sample size, residual imbalance, incomplete safety ascertainment, and short follow-up. Prospective randomized studies are required before ABT intensification can be recommended as a routine initial treatment strategy in this population.

## Data Availability

The original contributions presented in the study are included in the article/**Supplementary Material**. Further inquiries can be directed to the corresponding author.

## Supplementary material

Supplementary Figure 1 Covariate balance diagnostic before and after propensity score matching (Love plot). Absolute standardized mean differences (SMDs) are shown for all reported baseline characteristics before and after matching. For covariate balance assessment, baseline HIV RNA was log10-transformed. Red circles represent SMDs before matching, and green triangles represent SMDs after matching. The vertical dashed line indicates an absolute SMD of 0.10. Substantial residual imbalance remained after matching, particularly for education level, baseline HIV RNA, route of infection, baseline CD4+ T-cell count, ethnicity, and sex.

Supplementary Figure 2 Propensity score density distribution before and after matching. Density plots show the fitted propensity score distributions for the ABT+B/F/TAF and B/F/TAF groups before and after matching. Before matching, the two groups showed a broad region of common support, although their propensity score distributions differed. After matching, propensity score overlap improved substantially. The post-matching distributions were based on the unweighted matched sample.

Supplementary Table 1 Absolute standardized mean differences before and after propensity score matching
