# Supplementary material for: Adjunctive albuvirtide is associated with improved immune recovery in treatment-naive patients with advanced HIV disease receiving bictegravir/emtricitabine/tenofovir alafenamide: a retrospective propensity score-matched cohort study
Source: Front Immunol. 2026 Aug 10;17:1900068. doi: 10.3389/fimmu.2026.1900068 (PMC13500597; doi:10.3389/fimmu.2026.1900068)
Supplement: Supplementary file 1 [file SupplementaryFile1.docx]

**Figure S1. Covariate balance diagnostic before and after propensity score matching (Love plot).**

Absolute standardized mean differences (SMDs) are shown for all reported baseline characteristics before and after matching. For covariate balance assessment, baseline HIV RNA was log10-transformed. Red circles represent SMDs before matching, and green triangles represent SMDs after matching. The vertical dashed line indicates an absolute SMD of 0.10. Substantial residual imbalance remained after matching, particularly for education level, baseline HIV RNA, route of infection, baseline CD4+ T-cell count, ethnicity, and sex

.

**Figure S2. Propensity score density distribution before and after matching.** Density plots show the fitted propensity score distributions for the ABT+B/F/TAF and B/F/TAF groups before and after matching. Before matching, the two groups showed a broad region of common support, although their propensity score distributions differed. After matching, propensity score overlap improved substantially. The post-matching distributions were based on the unweighted matched sample.

**Supplementary Table S1. Absolute standardized mean differences before and after propensity score matching**

| **Variable** | **SMD_Before** | **SMD_After** |
| --- | --- | --- |
| Marital_Married | 0.020152 | 0.057636 |
| Marital_Unmarried | 0.020152 | 0.057636 |
| Baseline_CD4_CD8 | 0.030712 | 0.087597 |
| Age | 0.066319 | 0.092825 |
| BMI | 0.085153 | 0.128505 |
| Education_High | 0.105605 | 0.032669 |
| WHO_stage_3 | 0.137102 | 0.018538 |
| Route_Homosexual | 0.146659 | 0.247600 |
| Route_Uncertain | 0.185851 | 0.187549 |
| Sex_Female | 0.193737 | 0.194390 |
| Sex_Male | 0.193737 | 0.194390 |
| Route_Heterosexual | 0.201045 | 0.273954 |
| Route_IDU | 0.208514 | 0.250000 |
| Baseline_CD4 | 0.308284 | 0.238513 |
| Ethnicity_Han | 0.266903 | 0.214898 |
| Ethnicity_Other | 0.266903 | 0.214898 |
| WHO_stage_2 | 0.310187 | 0.164824 |
| Days_to_tx_<=7.0 | 0.427446 | 0.010714 |
| Days_to_tx_>7.0 | 0.427446 | 0.010714 |
| WHO_stage_4 | 0.448686 | 0.131329 |
| Baseline_HIV_RNA_log10 | 0.414672 | 0.414544 |
| Education_Middle | 0.614365 | 0.609771 |
| Education_Bachelor | 0.620256 | 0.446421 |
| Comorbidities_0 | 0.642732 | 0.105280 |
| Comorbidities_1 | 0.642732 | 0.105280 |
| Education_Unknown | 1.643168 | 1.371989 |

Note: SMD, standardized mean difference; baseline HIV RNA was log10-transformed.
